# Methodological bias shapes the interpretation of the urinary microbiome in low-biomass systems

**DOI:** 10.3389/fmicb.2026.1851926

**Published:** 2026-07-30

**Authors:** Viviana Méndez-Sacta, Solmayra Agreda Orellana, Luz María Ayavaca-Tapia, Yomaira Yolanda Gutiérrez León, William Calero-Cáceres

**Affiliations:** 1 Faculty of Medical Sciences, University of Cuenca, Cuenca, Ecuador; 2 Biotechnology Program, Department of Food and Biotechnology Science and Engineering, Universidad Técnica de Ambato, Ambato, Ecuador; 3 UTA-RAM-One Health, Group for Universal Advance in Bioscience, Department of Food and Biotechnology Science and Engineering, Universidad Técnica de Ambato, Ambato, Ecuador

**Keywords:** clinical microbiology, low-biomass microbiome, methodological bias, microbiome analysis, multi-omics, urinary microbiome, urobiome

## Abstract

The recognition of the urinary microbiome (urobiome) has challenged the long-standing paradigm of urinary tract sterility, revealing diverse microbial communities associated with both urinary health and disease. However, the characterization of the urobiome remains highly variable across studies, particularly due to the low-biomass nature of urine samples and the strong influence of methodological bias. In this Mini Review, we examine how factors such as sample collection methods, contamination dynamics, DNA extraction protocols, sequencing approaches, and bioinformatic pipelines shape microbial profiles and contribute to inconsistencies in microbiome–disease associations. We further discuss the limitations of traditional culture-based diagnostics, which fail to detect many microorganisms identified through sequencing-based approaches, and highlight the need for standardized, contamination-aware, and function-oriented frameworks. Finally, we explore the potential of integrated multi-omics strategies to improve the reliability, reproducibility, and clinical relevance of urobiome research in urinary tract health and disease.

## 1 Introduction

For decades, the urinary tract was considered a sterile environment in the absence of infection, a paradigm largely shaped by the limitations of conventional culture-based microbiology. The development and application of culture-independent techniques have challenged this view, demonstrating that urine can harbor diverse and dynamic microbial communities, collectively referred to as the urobiome (Pastuszka et al., 2025; Robino et al., 2025). With the advancement of culturing techniques, such as expanded quantitative urine culture (EQUC), and genomic approaches, including 16S rRNA gene sequencing for bacterial profiling and internal transcribed spacer (ITS) sequencing as the primary marker for fungal characterization, microorganisms have been detected not only in individuals with urinary tract infections but also in asymptomatic populations, redefining the urinary tract as a biologically active microbial niche rather than a sterile compartment (Pearce et al., 2014; Price et al., 2016; Deen et al., 2023; Sohail et al., 2025).

Despite these advances, the characterization of the urobiome remains highly variable across studies. Reported microbial profiles from urine samples differ not only according to host-related factors — such as sex, hormonal status, age, and anatomical differences—but also according to methodological approaches, including sample collection strategies and sequencing techniques (Hrbáček et al., 2021; Kool et al., 2023). In addition, many microorganisms detected through sequencing-based approaches are not recovered by standard clinical culture, highlighting the limitations of traditional diagnostic frameworks and suggesting that a substantial fraction of the urinary microbiota has historically remained undetected (Baimakhanova et al., 2025; Bermudez et al., 2025).

These limitations have important implications for microbiological interpretation and clinical translation. Increasing evidence suggests that microbial community structure may be associated with urinary tract conditions, including urinary tract infections, urinary incontinence, overactive bladder, and interstitial cystitis (Neuhaus et al., 2022; Zheng et al., 2023; Dickson et al., 2024). However, the consistency and biological meaning of these associations remain uncertain, particularly when methodological variability is not explicitly accounted for. A critical question therefore emerges: to what extent do current methodologies capture true biological signals, and to what extent do they introduce systematic bias?

In this Mini Review, we examine how methodological bias shapes microbial detection and ecological interpretation in urobiome research. We further discuss the consequences of this variability for microbiological interpretation and clinical translation and consider directions toward more standardized and functionally informed analytical frameworks.

## 2 Methodological constraints in low-biomass urobiome studies

### 2.1 The urobiome as a low-biomass microbial system

Low-biomass microbial systems are characterized by a narrow dynamic range, where measured signals can approach the limits of detection and therefore become disproportionately shaped by methodological design (Austin and Korem, 2024). In this setting, the sensitivity of next-generation sequencing (NGS) is a double-edged feature: while it enables detection of low-abundance nucleic acids, it simultaneously increases the likelihood that contaminant sequences are sampled and retained within the final read set, thereby amplifying signals that may not originate from the biological specimen (Erb-Downward et al., 2020). When microbial loads are low, contaminant DNA can dominate sequencing outputs, distorting apparent diversity and community structure (Goig et al., 2020). Consequently, urinary studies must be interpreted as observations emerging from a coupled system—biology and measurement—rather than as direct readouts of a stable, self-contained microbial community.

Analogous interpretive challenges are well documented in other low-biomass niches, where community profiles can be strongly influenced by sensitivity to non-biological input. For example, lung microbiome studies have highlighted how low bacterial loads and contamination-prone sampling can shape inferred microbial communities (Marsh et al., 2018; Selway et al., 2020). Similarly, placenta microbiome research has underscored the risk that microbial signals may be indistinguishable from contamination controls, even when sophisticated workflows are used (Lauder et al., 2016; Gschwind et al., 2020; Panzer et al., 2023). In the case of the urobiome, these issues are compounded by anatomical proximity to high-biomass sites, which increases the probability that external signals enter low-biomass urine measurements (Reasoner et al., 2025). Taken together, technical variability in urobiome studies is not a secondary nuisance that can be fully “averaged out” after sequencing; instead, it acts as a structural constraint that determines how biological narratives can be inferred from urine-based data.

### 2.2 Sources of methodological bias across the analytical workflow

Methodological bias arises at each stage of the urobiome workflow, from sample collection to bioinformatic analysis, and plays a defining role in how microbial communities are detected and interpreted. As a result, methodological choices can independently shape which microbial signals are observed and how they are represented. Even when biological specimens are nominally comparable, differences in analytical implementation can yield divergent representations of the same underlying biological material.

A primary source of variability is sampling methodology, which directly determines the microbial fraction being analyzed. Comparative studies have shown that voided, midstream, and catheterized urine samples yield distinct microbial profiles, with differences in both alpha and beta diversity associated with collection strategy (Pohl et al., 2020; Hrbáček et al., 2021). Voided samples frequently include contributions from urethral and periurethral microbiota, whereas catheterized samples reduce, but do not eliminate external contamination (Ozer et al., 2021). These differences are not fully correctable through downstream computational approaches, indicating that sampling bias cannot be resolved *post hoc*.

Contamination from laboratory reagents and workflows represents an additional and well-documented source of bias in low-biomass microbiome studies (Salter et al., 2014; Calero-Cáceres et al., 2024; Rauer et al., 2025). DNA present in extraction kits and PCR reagents varies across batches and can substantially influence sequencing outputs, and in low-biomass samples contaminant sequences may constitute a substantial fraction of total reads, leading to erroneous detection of taxa and inflated diversity estimates (Weyrich et al., 2019; Lai et al., 2025). Recent multi-laboratory analyses also indicate that contamination remains a persistent challenge even with increasing awareness (Anwar et al., 2025). Therefore, systematic inclusion of negative controls and contamination-aware analytical frameworks are essential.

A further limitation stems from the reliance on DNA-based detection, which does not distinguish between viable microorganisms, dead cells, and extracellular DNA (Rogers et al., 2010; Corrales-Martinez et al., 2022). This limitation is relevant in the urinary tract because residual microbial DNA may persist following antimicrobial treatment, such that sequencing detection does not necessarily indicate biological activity or clinical relevance (Emerson et al., 2017; Lee et al., 2022). Approaches such as propidium monoazide treatment have been proposed to address this issue, but their performance remains inconsistent and strongly dependent on sample composition and biomass (Kaur et al., 2025). Consequently, detection of microbial DNA cannot be directly interpreted as evidence of active colonization.

Sequencing strategy introduces an additional source of methodological divergence. Metabarcoding approaches, including 16S rRNA gene sequencing for bacterial profiling and internal transcribed spacer (ITS) sequencing for fungal characterization, can generate substantially different microbial profiles compared with shotgun metagenomics due to primer bias, amplification efficiency, and differences in taxonomic resolution (Villette et al., 2021; Heidrich et al., 2022; Lamoureux et al., 2022). While shotgun metagenomics can provide higher taxonomic resolution and characterization of microbial functional potential, it is more susceptible to host DNA interference and requires greater sequencing depth, which can limit its applicability in low-biomass environments (Lewis et al., 2025; Plaza Oñate et al., 2025). As a result, findings generated using different sequencing approaches are not directly comparable without careful consideration of methodological context (D’Amore et al., 2016; Han et al., 2020).

Bioinformatic processing introduces an additional layer of variability. Differences in reference databases such as SILVA, Greengenes, and RDP can lead to inconsistent taxonomic assignments and variability in diversity metrics, even when identical datasets are analyzed (Roume et al., 2023; Molano et al., 2024; Domingues et al., 2025). In addition, incomplete reference genomes and annotation errors further complicate accurate identification of microbial taxa and functional genes, including antimicrobial resistance determinants (Balvočiute and Huson, 2017; Doyle et al., 2020) Together, these issues mean that computational design can directly shape the biological conclusions drawn from the same underlying sequencing reads. Differences observed across studies may therefore reflect variations in analytical design as much as underlying microbial ecology, fundamentally limiting the reproducibility and comparability of urobiome findings because methodological choices across multiple workflow stages can independently reshape the microbial profiles reported (Cumpanas et al., 2020). Urobiome interpretation must consequently be situated in the context of the full analytical workflow rather than treating results as directly comparable across studies.

### 2.3 From microbiological uncertainty to clinical implications

The absence of a consistent core urinary microbiota or urobiota means that different studies have reported distinct dominant taxa and community structures (Cumpanas et al., 2020; Ammitzbøll et al., 2021; Reasoner et al., 2025). Rather than reflecting only biological diversity, this instability is also consistent with methodological effects that shape which microbial fraction is detected and how it is classified. Conceptually, urine can be viewed as a dynamic compartment in which flow, dilution, and voiding dynamics continuously reshape the microbial landscape, unlike more static high-biomass niches such as stool; this dynamism plausibly compounds interpretive uncertainty when study designs differ (Huttenhower et al., 2012; Neugent et al., 2020; Wang et al., 2026).

Clinical studies face a related challenge: apparent overlap between symptomatic and asymptomatic groups can be substantial, limiting the use of any single dominant taxon as a stable biomarker (Adu-Oppong et al., 2022; Hochstedler-Kramer et al., 2022). Dysbiosis, in turn, remains inconsistently defined across studies, with differing baselines and thresholds that reduce reproducibility and constrain predictive value (Hooks and O’Malley, 2017; Tiffany and Bäumler, 2019; Kelley, 2024).

These biological ambiguities translate into diagnostic uncertainty. Conventional urine culture remains poorly suited to capturing urobiome-relevant complexity because it favors fast-growing organisms and frequently misses polymicrobial or fastidious taxa (Szlachta-McGinn et al., 2022; Sathiananthamoorthy et al., 2023). In practice, clinically relevant signals can be misclassified as contamination, contributing to a translational gap between microbiome research and clinical expectations. Culture-independent approaches further complicate interpretation because they cannot reliably distinguish viable from nonviable organisms, meaning detected DNA may not correspond to active infection or clinically meaningful microbial processes (Janes et al., 2022; Szlachta-McGinn et al., 2022; Moreland et al., 2025).

Methodological bias can then amplify perceived group differences (Kim et al., 2022; Forry et al., 2024). Even when statistically significant taxa or community patterns are reported, differences may reflect analytical design—such as detection sensitivity, reference assignment, and low-biomass stochasticity—rather than biological divergence between patient phenotypes (Isali et al., 2024; Lewis et al., 2025). Collectively, the studies summarized in Table 1 demonstrate that methodological variability can arise at every stage of the urobiome analytical workflow, from urine collection and sample processing to sequencing and bioinformatic interpretation.

**Table 1:** Methodological factors influencing urobiome characterization and interpretation.

Workflow stage	Variable/approach	Representative studies	Main finding/interpretation
Urine collection	Voided midstream vs. catheterized bladder urine; first-catch vs. midstream vs. catheterized urine; cystoscopy urine; cystocentesis vs. voided urine; collection device/protocol	Southworth et al. (2019), Hourigan et al. (2020), Pohl et al. (2020), Hrbáček et al. (2021), and Coffey et al. (2023)	Collection method can substantially alter richness, alpha/beta diversity, and detected taxa. Voided samples often capture periurethral/urogenital contamination, whereas catheterized or bladder-access samples may better reflect bladder-associated communities.
Storage and preservation	Storage temperature, storage time, preservative use, time-of-day/day-to-day collection	Bundgaard-Nielsen et al. (2020) and Kumar et al. (2023)	Storage conditions and preservatives influence microbial stability and detection of low-abundance taxa. Standardization of post-collection handling is essential to improve reproducibility.
Urine volume and pre-processing	Processed volume; centrifugation speed/time; pelleting conditions	Ackerman et al. (2019) and Lewis et al. (2025)	Urine volume and pre-processing conditions affect DNA yield, microbial recovery, and downstream sequencing performance, especially in low-biomass samples.
DNA extraction	Extraction kit choice; lysis chemistry; protocol modification	Karstens et al. (2021), Mrofchak et al. (2021), and Zheng et al. (2025)	Extraction methods can produce different microbial profiles through differential recovery efficiency, reagent contamination, and lysis bias.
Host DNA depletion	Host depletion strategies for shotgun metagenomics	Lewis et al. (2025)	Host DNA depletion improves microbial recovery in shotgun approaches but adds workflow complexity and may influence community representation.
Amplicon choice and 16S region	16S rRNA target region; single-amplicon vs. multi-amplicon reconstruction	Heidrich et al. (2022) and Siddiqui et al. (2022)	Primer choice and amplicon region strongly influence taxonomic resolution and detected community structure; 16S profiling is restricted to bacterial community analysis.
Sequencing strategy	16S rRNA gene sequencing vs. shotgun metagenomics	Moustafa et al. (2018) and Lewis et al. (2025)	16S is lower-input and more tractable for broad bacterial profiling, whereas shotgun metagenomics offers higher resolution and functional inference but is more sensitive to host DNA interference and sequencing-depth requirements.
Detection platform	Culture-based methods vs. sequencing-based methods	Coba et al. (2019), Pohl et al. (2020), and Hrbáček et al. (2021)	Culture and sequencing are complementary but not interchangeable. Each detects different fractions of the urobiome and is affected by different biases.
Bioinformatic pipeline	QIIME 2 vs. NG-Tax vs. Mothur; OTU-based vs. ASV-based processing; clustering/filtering thresholds	Ducarmon et al. (2020) and Siddiqui et al. (2022)	Choice of pipeline and downstream analytical settings influences diversity estimates, clustering, and interpretability of results.
Reference database	SILVA vs. Greengenes vs. NCBI 16S reference sets	Heidrich et al. (2022)	Database choice can alter species-level assignment and comparative interpretation, underscoring the need for transparent and harmonized annotation frameworks.

## 3 Toward standardized and functionally informed urobiome research

Sample collection standardization is the highest-leverage intervention in urobiome research because it determines the biological material entering every downstream step of the workflow; computational harmonization cannot rescue profiles generated from inconsistent collection methods. Comparative work shows that voided, midstream, and catheterized urine yield distinct microbial compositions (Pohl et al., 2020; Hrbáček et al., 2021). Catheterized sampling reduces external contamination but does not eliminate it (Ozer et al., 2021). For research-grade studies, catheterized collection should be preferred when ethically and clinically feasible, whereas voided sampling remains more practical for large-scale settings. Minimum urine volume thresholds and explicit documentation of the collection route and protocol should be mandatory reporting standards to improve interpretability (Brubaker et al., 2021; Lewis et al., 2025; Reasoner et al., 2025).

In low-biomass urine, metabarcoding approaches remain the most tractable first-line option because they require substantially less input material and are less constrained by host-DNA interference than shotgun metagenomics (Ranjan et al., 2015; Zuo et al., 2022). However, methodological choices during library preparation and sequencing, including primer and target-region selection, amplification conditions, sequencing depth, and platform choice, can substantially influence microbial recovery and taxonomic resolution (Abellan-Schneyder et al., 2021; Villette et al., 2021; Heidrich et al., 2022). Shotgun metagenomics can provide higher taxonomic resolution and direct functional inference, but it becomes practically feasible only when sufficient urine volume is available and when host-DNA depletion strategies are used to mitigate the strong host-background signal (Matchado et al., 2024; Lewis et al., 2025). Consequently, standardized workflows incorporating appropriate controls, mock communities, and transparent reporting of sequencing parameters are essential to improve reproducibility and cross-study comparability (Eisenhofer et al., 2019; Abellan-Schneyder et al., 2021; Forry et al., 2024). A pragmatic strategy is therefore a hybrid design: use metabarcoding approaches to characterize broad community structure across cohorts and apply shotgun metagenomics to selected specimens where urine volume and sequencing depth support robust microbial recovery.

Contamination-aware analysis should be treated as mandatory rather than optional in urobiome studies. Sequencing in low-biomass urine is particularly vulnerable to contaminant DNA from reagents, laboratory environments, and sample processing, which can constitute a substantial proportion of the detected signal (Karstens et al., 2019; Yoon et al., 2025). Consequently, negative controls at the extraction, amplification, and library-preparation stages should be incorporated into every study design, with explicit reporting of how those controls inform downstream filtering decisions (Calero-Cáceres et al., 2024). Even workflows designed to account for contamination can generate divergent results across sites, reinforcing the need for harmonized reference materials and shared mock communities (Sinha et al., 2017; Han et al., 2020; Forry et al., 2024).

While contamination-aware workflows are essential to improve data reliability, future progress will also require moving beyond descriptive taxonomic profiling toward functional characterization. Both metabarcoding and shotgun metagenomics provide information on genetic potential, but they do not fully capture microbial activity, gene expression, or metabolite output (Kim et al., 2022). Multi-omics integration — combining metagenomics with metatranscriptomics, metabolomics, and culture-based validation — provides a route toward connecting microbial composition to function and clinical relevance (Aguiar-Pulido et al., 2016; Chetty and Blekhman, 2024; Matchado et al., 2024; Kumar et al., 2026). However, application in low-biomass urine is technically demanding: required input volumes and achievable sequencing depth often limit multi-omics feasibility under routine sampling conditions, and protocols tolerant of these constraints require further development (Kim et al., 2022; Lewis et al., 2025; Mukherjee et al., 2026).

Host-associated and environmental metadata are an underused lever for improving cross-study interpretability. Factors such as age, sex, hormonal status, comorbidities, and antibiotic exposure are likely to influence urinary microbial composition in complex and context-dependent ways, yet they are not consistently incorporated into study designs or analytical frameworks (Sawhney et al., 2021; Neugent et al., 2022; Kelly et al., 2024; Zou et al., 2024). Without harmonized minimum metadata standards, it remains difficult to separate host-driven variation from methodological artefacts and to translate findings across populations. A minimum reporting standard covering sex, age, menopausal status, recent antibiotic use, and urinary diagnosis would materially improve comparability (Govender et al., 2019; Adebayo et al., 2020; Nickel et al., 2022; Zou et al., 2024).

Beyond methodological harmonization, standardization efforts should also account for the biological characteristics that distinguish the urobiome from other low-biomass ecosystems (Govender et al., 2019; Perez-Carrasco et al., 2021; Lewis et al., 2025; Tiwary et al., 2026). Unlike invasive compartments, urine enables non-invasive longitudinal sampling within the same individual, allowing within-subject baselines to support interpretation. The urobiome is also directly linked to common, high-burden clinical conditions — urinary tract infection, urinary incontinence, overactive bladder, and interstitial cystitis — providing a clear translational rationale for methodological improvement (Neuhaus et al., 2022; Zheng et al., 2023; Dickson et al., 2024). Urinary microbial loads and community composition vary systematically with sex, hormonal status, and urological condition, enabling stratified analyses (Adebayo et al., 2020; Zou et al., 2024).

**Figure 1:**
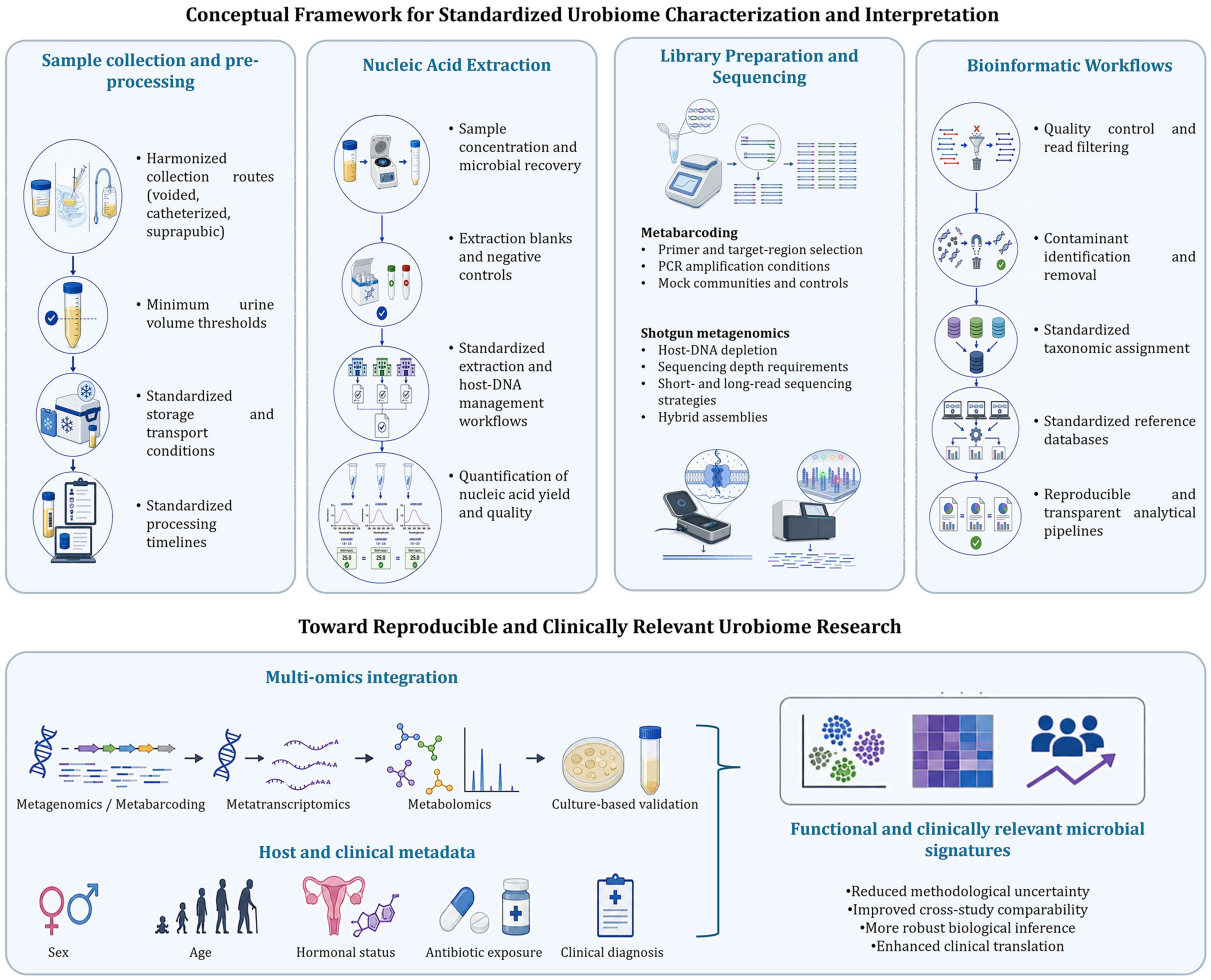
Conceptual framework for standardized and functionally informed urobiome research. Standardization of pre-analytical procedures, nucleic acid extraction, library preparation, sequencing, and bioinformatic workflows provides the foundation for reproducible urobiome characterization. Integration of multi-omics approaches with host and clinical metadata enables the identification of functional and clinically relevant microbial signatures, improving biological interpretation, cross-study comparability, and clinical translation.

Collectively, the evidence reviewed here suggests that improving reproducibility alone will not be sufficient to advance the field. Standardization must be accompanied by functional characterization and contextual interpretation to distinguish methodological variability from true biological signals. As summarized in Figure 1, a framework integrating harmonized analytical workflows, multi-omics approaches, and host-associated metadata may provide a pathway toward the identification of functional and clinically relevant microbial signatures within the urobiome.

Future progress will depend on integrating methodological rigor with biological interpretation across the entire workflow. Without coordinated efforts spanning sampling standardization, contamination-aware analysis, functional multi-omics integration, and consistent host metadata reporting, studies may remain technically reproducible yet biologically ambiguous (Brubaker et al., 2021; Cole et al., 2023). Shared reference materials, mock communities, and harmonized analytical workflows are a critical step toward enabling meaningful cross-study comparisons and reducing divergence attributable to analytical design (Kachroo et al., 2021). Until methodological variability is explicitly accounted for, the urobiome will remain a partially resolved system shaped as much by analytical design as by underlying biological reality.
